# Application effect of task-oriented multi-dimensional nursing intervention in patients with coronary atherosclerotic heart disease and concurrent hypertension

**DOI:** 10.4314/ahs.v24i3.41

**Published:** 2024-09

**Authors:** Weihe Li

**Affiliations:** Department of Nursing of Cardiology, The First Affiliated Hospital of Harbin Medical University, Harbin, China

**Keywords:** Coronary atherosclerotic heart disease, task-oriented theory, multi-dimensional nursing care, clinical nursing

## Abstract

**Background:**

The purpose of this study was to explore the application effect of task-oriented multi-dimensional nursing intervention in patients with coronary atherosclerotic heart disease (CAD) and concurrent hypertension.

**Methodology:**

The clinical data of 196 patients with CAD and concurrent hypertension in our hospital between January 2019 and January 2022 were retrospectively analysed. The patients receiving task-oriented multi-dimensional nursing were set as study group (n=98), while those given routine nursing as control group (n=98). The two groups were compared in the Hamilton Anxiety Scale (HAMA) scores, left ventricular end-diastolic diameter (LVEDD), 36-Item Short Form Health Survey (SF-36) scores, left ventricular ejection fraction (LVEF), Hamilton Depression Scale (HAMD) scores, systolic blood pressure (SBP), treatment compliance and incidence rates of adverse events.

**Results:**

After intervention, study group showed lower HAMA and HAMD scores, SBP and LVEDD, but higher SF-36 scores and LVEF than control group (P<0.05). The treatment compliance rate was higher in control group than that in study group (92.86% vs. 80.61%), while an opposite result was detected in the total incidence rate 5.10% vs. 13.27%, P<0.05).

**Conclusion:**

Multidimensional nursing benefits CAD patients with hypertension, improving emotions, compliance, and quality of life, reducing adverse events, and promoting recovery. Recommended for clinical use.

## Introduction

Coronary atherosclerotic heart disease (CAD) is a relatively common chronic wasting disease in the Department of Cardiology with the features of rapid onset, long course of disease and high mortality rate^1^,^2^. Cross-impact mechanism exists between CAD and hypertension. CAD complicated with hypertension, which is common in clinics, can accelerate the disease progression in patients with CAD, heighten the risk of cardiovascular injuries, and severely weaken the prognosis of patients^3^. Patients with CAD and concurrent hypertension face severe threats due to the refractory nature of the disease, so inadequate health management may result in recurrences, protractions, or progressions, and it increases the economic burden of patients by causing cerebrovascular accidents, heart failure, renal damage, retinal hypofunction, and multiple complications^4^. Therefore, clinicians should pay special attention to exploring scientific and effective nursing schemes, so as to reduce the risk of complications in patients with CAD and concurrent hypertension and increase their treatment compliance and recovery effect. Initially applied in clinical teaching, task-oriented theory has been used by some scholars^5^ in clinical nursing for patients in recent years, which is positively significant for increasing nursing management efficiency, staff s enthusiasm and nursing quality and decreasing nursing errors and disease risks. Task-oriented theory is a leadership theory that focuses on the relationship between the leader, the followers, and the tasks at hand. According to this theory, effective leaders are those who are able to analyse the task requirements and provide the necessary guidance and support to help their followers accomplish the task successfully. Task-oriented theory has been applied to nursing leadership to help guide nursing practice and promote high-quality patient care. In nursing, task-oriented leadership focuses on providing structure, organization, and clear expectations to ensure that patient care is delivered in a consistent and efficient manner. The primary focus of task-oriented nursing leadership is to accomplish tasks and meet goals while ensuring that patients receive safe and effective care. At present, most studies have focused on the task-oriented theory in teaching and training nurses in China, with few studies on the theory in disease nursing and no clear specific operation methods and effects. In the present study, the application effect of task-oriented multi-dimensional nursing intervention in patients with CAD and concurrent hypertension was explored as follows:

## Patients and methods

### Patients

The cross-sectional study method was adopted. From January 2019 to January 2022, 196 patients with CAD and concurrent hypertension were retrospectively analysed. The patients from July 2021 to January 2022 after the implementation of the task-oriented multi-dimensional nursing scheme were set as study group (n=96), while those from January 2019 to June 2021 before the scheme implementation as control group (n=98). Inclusion criteria were set as follows: 1) patients conforming to the diagnosis criteria for CAD^6^, the patients were >18 years old; 2) those in line with the diagnosis criteria for hypertension7, and 3) those who had complete clinical data and were fully informed of the nursing intervention content. Exclusion criteria were defined as follows: 1) patients with concurrent organic brain trauma, 2) those complicated with congenital coagulation dysfunction, 3) those with concurrent acute/chronic infection, 4) those with concomitant rib fracture or pneumothorax, 5) those with concurrent mental illness, or 6) those complicated with chest trauma, congenital heart defect or other diseases which may cause abnormal cardiac functions.

## Methods

The patients in control group received routine nursing: the patients and their family members were instructed to receive relevant examinations, and healthy education on the knowledge of CAD and hypertension treatment was conducted for patients. Psychological counseling was carried out for patients to relieve their fear about diseases and negative emotions. The nursing staff taught the patients routine self-measurement of cardiac function and blood pressure, strengthened the education on the self-health management of CAD and hypertension and raised their awareness of health management. Moreover, the contact methods of patients were collected so as to conduct post-hospital transitional care and health guidance, and the patients were told to take medicines as the doctor advised and return the hospital regularly for re-examination.

Task-oriented multi-dimensional nursing was conducted in study group: I. Construction of nursing group: a 4-6-member task-oriented nursing group was established with the head nurse of our department as the leader. Before the nursing scheme was officially performed, the group leader first systemically trained the group members to collect, sort and summarize risk factors of CAD with concurrent hypertension and evidence-based nursing measures by case study, literature analysis and experience sharing.

II. Establishment of task system: Several nurses carried out nursing interventions on each patient with CAD and concurrent hypertension by the shift system, namely, implementing their target tasks. Besides, the daily target tasks should be clarified in nursing work, and the completion status of these tasks should be detailed in the paper forms.

III. Clarification of multi-dimensional nursing tasks: 1) Diet care: the nurses provided healthy diet guidance for the patients in the hospital, recorded the patient's daily diet care tasks and task completion status, including food types, rough value of food energy, and proportions of three major nutrients (sugar, fat, and protein), and regulated the diet standard, mainly including easily digestive, high-fiber, light liquid food and no low-fiber, high-fat, high-sugar non-liquid food and spicy and irritating food.

2) Psychological nursing: the nurses actively communicated with patients to popularize the knowledge of CAD and concurrent hypertension towards patients and conducted psychological counseling for those with fear and anxiety. Besides, the group members accompanied the patients to receive diagnosis, examination and treatment, distracted the patients' attention by small talks with them, avoided leaving them alone for a long time, and recorded the psychological intervention time and content. 3) Social support: on the day of admission, the nurses should communicate with the patients' family members or friends, and ask them to accompany the patients during surgical treatment, if possible, avoid frequently talk with the patients about the disease- or treatment-related negative information, such as CAD or hypertension complications, treatment costs and other topics, and provide them with benign social support through positive guidance, thereby relieving anxiety of the patients.

4) Prevention and control of risk factors: high-sugar or high-fat diet, insufficient water intake, smoking, and sitting or lying for a long time are risk factors for CAD and hypertension, and improper control of these factors may decrease the quality of prognosis in patients with CAD and concurrent hypertension. Therefore, the above risk factors should be strictly controlled in the process of treatment and rehabilitation. The patients were instructed to moderately drink and exercise and scientifically intake food according to the doctor's advice every day.

The completion of the task was recorded in the daily task form.’ body recovered to meet the training conditions, the patients were guided to carry out rehabilitation exercise to promote the rehabilitation of cardiac function with the permission of the attending physician specifically as follows: after completing 5 min of slow walking (speed: 1.5-2.5 km/h) for warm-up training, 5 min of fast walking training (speed: 4.0-5.5 km/h), and 15 min of jogging training (speed: 6.0-7.5 km/h) (all training speed was adjusted according to the patient's limb motor function, and the fastest speed that the patients could tolerate was appropriate), the patients were guided to receive routine training of finger, wrist, elbow, shoulder, knee, ankle, and toe joint motion and basic training of in-situ pacing, slow walking, jogging, in-situ squats, and upward leg swinging for 15-20 min/d.

IV. Evaluation of task effect: all the detailed work in the content of the nursing task needs to be “daily completed” during the implementation period, that is, the corresponding work tasks should be completed with quality and quantity every day, and at the same time, the completion time of each task and the index parameters to be monitored need to be recorded, so as to evaluate the work efficiency and quality of the nurses. Task effect was mainly assessed through the establishment of “nursing task evaluation form”. The daily work quality of nurses was evaluated from two aspects of work completion time and work completion quality according to the actual completion of the nursing process, and tasks not completed accurately (not completed, not completed on time, and not completed in accordance with the requirements of the daily completed task) were recorded faithfully. The corresponding nurse's task performance scores were deducted by 3 points, 2 points, and 1 point for not completed, not completed on time, and not completed according to the task requirements, respectively. The task performance score of each nurse was 100 points per month and subjected to the reduction system. The performance of each nurse was ranked according to the actual remaining scores at the end of each month, and the higher the remaining scores were, the better the work was completed.

V. Motivation mechanism for nurses: the performance was ranked weekly and monthly according to the task performance score of the nurses, and the top three nurses in the performance ranking were given the “weekly or monthly work hotshot award”, and the three rankings: “the first, second and third places” were set according to the actual rank of nurses. The winner was awarded direct material incentives through the distribution of bonus and gifts and other means. At the same time, the nurses with outstanding performance in nursing were praised in the hospital or the department and had the priority to training. All the nurses were encouraged to work actively and improve work efficiency through material motivation and spiritual motivation.

### Observation indicators

The Hamilton Anxiety Scale (HAMA) 8 scores, left ventricular end-diastolic diameter (LVEDD), the 36-Item Short-Form Health Survey (SF-36) 9 scores, left ventricular ejection fraction (LVEF), Hamilton Depression Scale (HAMD) 10 scores, systolic blood pressure (SBP), treatment compliance and the incidence rates of adverse events were compared between the two groups specifically as follows: I. Quality of life and mental status: the scores of HAMA (14 items), SF-36 (36 items) and HAMD (24 items) were compared between the two groups before intervention (T0) and at 2 months after intervention (T1), which were positively correlated with the degree of anxiety, quality of life and degree of depression, respectively. II. Disease-related indicators: the LVEDD, SBP and LVEF values were compared at T0 and T1 between the two groups. SBP was measured using an electronic sphygmomanometer and the other two indicators using a colour ultrasound diagnostic system. III. Treatment compliance: 1) poor: during treatment, the patient refused to receive examination and treatment or follow the doctor's advice, thereby delaying or changing related treatment or examination items, 2) good: although the patient refused to receive examination and treatment or follow the doctor's advice, such behaviour was timely terminated after intervention of medical staff or family members and no related treatment or examination items were delayed and changed, and 3) excellent: in the whole treatment process, the patient cooperated well, without non-compliance behaviour (excellent compliance rate = the number of patients with excellent compliance/the total ×100%). IV. Adverse events: The incidence rates of unstable angina, cardiac syncope, and heart failure were compared between the two groups.

### Statistical methods

Statistical Product and Service Solutions (SPSS) 22.0 (IBM, Armonk, NY, USA) was used for statistical analysis. Ranked data (hypertension grades and cardiac functional grades) were subjected to rank sum test, measurement data (HAMA and SF-36 scores, LVEDD, LVEF, SBP, age and course of disease) were analysed using t test, and χ^2^ test was adopted for enumeration data (the excellent compliance rate, incidence rates of adverse events, and sex). P<0.05 was considered to be statistically significant.

## Results

### Comparison of general data between the two groups

There was no significant difference in age (t = 0.801), CAD duration (t = 1.261), hypertension duration (t = 0.604), hypertension grade (Z = 0.409), cardiac functional classification (Z = 0.245), and gender (x^2^ = 0.898) between the two groups (P > 0.05), as shown in Table 1.

**Table 1:** Comparison of general data between the two groups

Group	N	Age (years)	CAD duration (months)	Duration of hypertension (months)	Hypertension		Cardiac function		Gender	

					Grade 1-2	Grade 3	Grade I-II	Grade III-IV	Male	Female
Study group	98	60.35 ± 4.15	6.41± 1.12	12.35 ± 2.15	69	29	75	23	58	40
Control group	98	59.89 ± 3.89	6.68 ± 1.80	12.17 ± 2.02	73	25	72	26	46	42
t/Z/x^2^		0.801	1.261	0.604	0.409		0.245		0.898	
P		0.424	0.209	0.547	0.522		0.621		0.343	

### Comparison of quality of life and psychological status between the two groups

At T0, there was no significant difference in HAMA (t = 1.545), SF-36 (t = 0.232), HAMD (t = 0.426) between the two groups (P > 0.05); at T1, Fig. HAMA (t = 14.223) and HAMD (t = 14.502) were lower and SF-36 (t = 5.275) was higher in the study group than in the control group (P < 0.05), as shown in Table 2.

**Table 2:** Comparison of quality of life and psychological status between the two groups (x̅±s, points)

Group	N	HAMA		SF-36		HAMD	

		T0	T1	T0	T1	T0	T1
Study group	98	29.40 ± 3.86	14.43 ± 1.59	63.85 ± 7.76	80.24 ± 9.22	35.14 ± 3.39	16.36 ± 1.71
Control group	98	28.56 ± 3.75	18.33 ± 2.20	64.11 ± 7.90	73.59 ± 8.41	34.93 ± 3.51	20.35 ± 2.12
t		1.545	14.223	0.232	5.275	0.426	14.502
P		0.124	0.000	0.816	0.000	0.671	0.000

### Comparison of disease-related indicators between the two groups

At T0, there was no significant difference in LVEF (t = 0.540), SBP (t = 0.707), LVEDD (t = 0.276) between the two groups (P > 0.05); at T1, LVEF (t = 6.633) was greater in the study group than in the control group, SBP (t = 15.109) and LVEDD (t = 7.942) were lower than the control group (P < 0.05), as shown in Table 3.

**Table 3:** Comparison of disease related indicators between the two groups (x± s)

Group	N	L VEF (%)		SBP (mmHg)	LVEDD mm		

		T0	T1	T0	T1	T0	T1
Study group	98	47.12 ± 3.11	55.53 ± 2.53	175.35 ± 3.62	118.45 ± 2.59	58.12 ± 4.63	50.20 ± 3.25
Control group	98	47.35 ± 2.85	52.46 ± 3.82	174.98 ± 3.71	124.43 ± 2.94	58.30 ± 4.51	54.31 ± 3.96
t		0.540	6.633	0.707	15.109	0.276	7.942
P		0.590	0.000	0.481	0.000	0.783	0.000

### Comparison of treatment compliance between the two groups

The excell ent compliance rate in the study group (92.86% vs. 80.61%) was higher than that in the control group (x^2^ = 6.386, P < 0.05), as shown in Table 4.

**Table 4:** Comparison of treatment compliance between the two groups [n (%)]

Group	N	Superior	Good	Poor	Compliance Excellence Rate
Study group	98	91 (92.86)	5 (5.10)	2 (2.04)	91 (92.86)
Control group	98	79 (80.61)	14 (14.29)	5 (5.10)	79 (80.61)
X^2^					6.386
P					0.012

### Comparison of adverse events between the two groups

The overall adverse event rate in the study group (5.10% vs. 13.27%) was lower than that in the control group (x^2^ = 3.915, P < 0.05), as shown in Table 5.

**Table 5:** Comparison of adverse events between the two groups [n (%)]

Group	N	Unstable angina pectoris	Cardiac syncope	Cardiac failure	Overall adverse event rate
Study group	98	3 (3.06)	0	2 (2.04)	5 (5.10)
Control group	98	6 (6.12)	2 (2.04)	5 (5.10)	13 (13.27)
X^2^					3.915
P					0.048

## Discussion

The pathogenesis of hypertension is complex and related to a combination of genetic, environmental, systemic diseases, dietary structure, living habits and other factors, which can easily cause complications such as hypertensive nephropathy, hypertensive heart disease, ischemic encephalopathy^11^. Moreover, hypertension can severely increase the burden on the circulation of patients and exacerbate vascular endothelial injury, thereby increasing the risk of various complications such as CAD and heart failure^12^. The prognosis for patients with CAD with hypertension is poor, difficult to treat and repetitive. Clinical patients are primarily manifested as elevated blood pressure, abnormal heart rate, chest crushing pain, generalized weakness, sweating and other symptoms, which may pose a serious risk of heart failure if not treated promptly^13^.

Therefore, it is important to strengthen the intensity of clinical intervention and optimize the intervention process for the physical rehabilitation of CAD patients with hypertension The basic nursing work in most Chinese hospitals is currently consolidated, but specialist nursing services are still not standardized and refined. Most hospitals do not yet have a comprehensive set of targeted nursing architecture in their cardiovascular medicine departments, which limits the improvement of medical services in hospitals in a certain way. Task-driven nursing can drive and maintain nurses' enthusiasm and achievement motivation through “setting nursing tasks and incentive mechanisms”, so as to improve the quality of clinical nursing care for CAD patients with hypertension and improve the prognosis and outcome of patients.

In this study, the study group receiving task-driven multi-dimensional nursing intervention had lower HAMA score and SBP and LVEDD levels after intervention than the control group receiving routine care, and higher SF-36 score than the control group, indicating that task-driven multi-dimensional nursing was superior to routine care in improving psychological adverse emotions and improving quality of life in CAD patients with hypertension, similar to the study by Song et al.^14^. The results also showed that the treatment compliance of patients in the study group was higher than that in the control group, and the incidence of cardiovascular adverse events was lower than that in the control group, indicating that task-driven multidimensional care can improve the treatment compliance of CAD patients with hypertension and reduce the risk of cardiovascular adverse events. To analyse the reasons for the differences in the above results, this is due to the continuous improvement of clinical patients and all sectors of society on the quality of care requirements, clinical specialist nurses face increasing work pressure, and the conventional nursing model is difficult to specific the specific work tasks to each person every day, so nurses often have a sense of urgency in the actual work process, which limits the improvement of nurses' enthusiasm and work efficiency; task-driven multidimensional nursing can refine and implement various tasks in CAD with hypertension care to each nurse, improving the standardization of nursing programs, while improving the nurses' nursing enthusiasm and work efficiency through daily records, periodic assessment, reward incentives, making diet care, social support, psychological care, risk factor prevention and control, cardiac rehabilitation training and other body, heart, spiritual dimension of evidence-based nursing measures can be properly implemented, improving the patients' psychological adverse emotions, avoiding the risk factors of CAD with hypertension treatment, promoting the recovery of patients' cardiac function, and improving the quality of prognosis.

## Conclusion

In summary, the application of task-driven multi-dimensional nursing in CAD patients with hypertension has a good effect, which can effectively improve the psychological adverse emotions of CAD patients with hypertension, improve treatment compliance and quality of life, promote physical rehabilitation of patients, reduce the risk of cardiovascular adverse events, and has the value of clinical promotion.

## Data Availability

The datasets used and analysed during the current study are available from the corresponding author on reasonable request.
